# Comparison of school day eating behaviours of 8–11 year old children from Adelaide, South Australia, and London, England: Child eating behaviours in South Australia and England

**DOI:** 10.3934/publichealth.2018.4.394

**Published:** 2018-10-25

**Authors:** Dorota Zarnowiecki, Meaghan S Christian, James Dollman, Natalie Parletta, Charlotte E.L Evans, Janet E Cade

**Affiliations:** 1 School of Pharmacy and Medical Sciences, University of South Australia; City East Campus, North Terrace, Adelaide, SA 5000; 2 School of Clinical and Applied Sciences, Faculty of Health and Social Sciences, Leeds Beckett University; City Campus, Calverley Street, Leeds, LS1 3HE, UK; 3 Nutritional Epidemiology Group, School of Food Science and Nutrition, University of Leeds; Woodhouse Lane, Leeds, LS2 9JT, UK; 4 Alliance for Research in Exercise, Nutrition and Activity, School of Health Sciences, University of South Australia; City East Campus, North Terrace, Adelaide, SA 5000; 5 Centre for Population Health, School of Health Sciences, University of South Australia; South Australian Health and Medical Research Institute (SAHMRI), North Terrace, Adelaide, SA 5000

**Keywords:** children, eating behaviours, lunchbox, school meal, vegetable intake, fruit intake, school food policy

## Abstract

**Objective:**

School food intake makes a considerable contribution to children's overall diet, especially fruit and vegetable intake. Comparing differing school food provision systems can provide novel insights for intervention and improved nutrition policy. This study compared school day food in children from Australia and England.

**Design:**

Children completed food frequency questionnaires reporting school day food intake, breakfast intake and family evening meals. Differences in food consumed over the school day between Australian and English children were evaluated. Multinomial logistic regressions compared fruit and vegetable intake, family dinner frequency and breakfast in Australian and English children adjusting for confounders: age, sex, ethnicity and parent education.

**Setting:**

27 Primary schools in Adelaide, Australia and 32 in London, England.

**Subjects:**

N = 772 children aged 8–11 years from the Australian REACH study (n = 347) and UK RHS School Gardening Trial in England (n = 425).

**Results:**

Considerably more English children reported consuming vegetables at school than Australian children (recess/lunchtime Australian children 3.4%/6.1%; English children recess/lunctime 3.6/51.1%). However, Australian children were more likely to consume vegetables daily (OR = 4.1; 1.3, 12.5), and have family evening meals everyday [OR = 4.01; 1.88, 8.55], and were less likely to consume breakfast (OR = 0.26; 0.08, 0.79) than English children.

**Conclusions:**

Findings indicate that provision of a school lunch meal, compared to a packed lunch from home, may be more supportive of children's vegetable intake. However, without a supportive home environment that encourages vegetable intake, children will not be able to consume sufficient amounts of vegetables.

## 1. Introduction

The impact of poor nutrition in children is causing public health concerns around the world, and contributing to rising childhood obesity [1],[2]. Diet plays a fundamental role in weight management, and it is vital to foster healthy dietary behaviours from a young age to establish long term healthy habits [1],[3],[4]. Recently Evans et al have explored how the policies around health promotion and provision of food during the school day are having a considerable impact on overall diet intake in children [5]. Two countries with considerably different school food provision practices are Australia and England. England school food provision is centred on the school providing a school meal, whereas in Australia, most children bring in their own meal prepared by their parents, a “packed lunch” [6]. Only one other study has considered how differing food provision practices affect children's overall nutritional intake in England and Australia [7]. This review highlighted considerable differences in school nutrition policies between Australian and England, noting potential nutritional advantages of school meal provision for improving diet quality in children. However, understanding of the potential impact of school nutrition policies for improving nutritional intake is limited by a lack of monitoring and evaluation of policy compliance and dietary intake. Another important finding was whilst during school hours children's food consumption is restricted to meet nutrition standards it is the home food environment that can have a lasting impact on overall diet quality [7].

In Australia and England, primary school children's diets are not meeting national recommendations, with a high prevalence of unhealthy eating patterns and inadequate intake of core foods [8]–[11]. British children's diets are deficient in vital nutrients such as fibre, long-chain fatty acids, iron and vitamin D [11]. Many British children consume excess energy dense foods including hot chips (French fries), biscuits and crisps [12]. It is predicted that by 2025, 25% of children aged 6 to 10 years in the England will be obese if dietary and physical activity behaviours do not improve [13]. Likewise, Australian children consume too much saturated fat, sugar, sodium, with excess energy provided from discretionary foods such as cakes, biscuits and pastries [10],[14],[15]. Approximately 50% of Australian 9–13 year old children consume 1–3 serves of fruit daily, and less than 20% consumed at least two serves of vegetables daily [14].

Breakfast, school meals and family meals are key times when children consume a majority of their food intake [7],[16]–[18], and therefore provide windows of opportunity for improving children's food intake. In particular, food during the school day can make a considerable contribution to the overall quality of a child's diet [19],[20]. Children spend approximately 6–7 hours of the day at school, and during this time consume approximately one-third of their daily food intake [21] across 2–3 eating occasions, including a morning snack break (“Crunch&Sip”—Australian primary schools [22]), recess and lunch time. School food provision practices vary between Australia and England. In England, primary school children have the option of bringing a packed lunch from home, or paying for a school meal. Under the current policy all children up to seven years are eligible for a free school meal, with 85% of families electing for children to receive a school provided meal rather than providing a packed lunch [23]. Beyond the age of seven, parents have to pay approximately £2 pounds/AU$3.50 per day, and the uptake in school lunches varies from 34 to 99 percent [23]. In Australia, school meals are not provided and most children bring a packed lunch to school [6],[24], but may purchase food from a school canteen. The school canteen or tuckshop can provide children with a snack, lunch or drink. The foods provided in these canteens are based on the Australia dietary guidelines and each State/Territory has their own polices [7]. The South Australian “Right Bite” canteen guidelines which were rolled out in 2008, utilise a traffic light system, whereby red foods that are high in fat, salt and sugars (i.e sugary drinks, confectionary deep fried foods, savoury snack foods etc) are restricted from sale in canteens, and amber foods (i.e. processed meats, savoury commercial products such as meat patties or sausages, full-fat dairy) are recommended to be limited [25]. Notably, beyond the banning of the red foods, individual schools are able to decide how they implement the guidelines and compliance is not monitored [26]. The food policies applied to schools in England are quite similar, however, policies apply to the school meal that children receive. The school meal must provide fruit and vegetables, with adequate provision of dairy, low fat protein, and low fat starchy foods. Similar to schools in South Australia, foods high in fats and sugars are restricted [7]. Thus comparison of children's food intake within these differing school food provision practices can provide insights for intervention targets and improving nutrition policy. This paper utilises data from two studies conducted in Australia and England, providing a unique opportunity to more directly compare findings across two countries with different school food provision policies. The aim of this study was to compare school day food intake and breakfast, lunch and family meal habits, in children from Australia and England.

## 2. Methods

This analysis combined data collected from two studies; the “Resilience in eating and activity for child health” (REACH) study conducted in Adelaide, Australia in 2010, and the Royal Horticultural Society (RHS) Project: “Can a school gardening intervention improve children's diets” study conducted in London, UK 2010. Data were collected in both studies using food frequency questionnaires that allowed for comparison of children's dietary intake and eating habits between studies. For this study, data from a total of 772 children aged between 8 to 11 years old from the REACH and RHS studies were included in the analysis, as described before [16],[27]. This study was conducted according to the guidelines laid down in the Declaration of Helsinki. Ethics approval for the REACH study was obtained from the University of South Australia Human Research Ethics Committee and the Department of Education and Children's Services Ethics Committee. Ethics approval for the RHS study was obtained through the Leeds Institute of Health Sciences and Leeds Institute of Genetics, Health and Therapeutic Joint Ethics Committee (Reference number: 09/012). Parents provided written consent for their family to participate in the study, and children provided verbal assent before commencing study measures.

### 2.1. REACH study methodology

REACH was a cross-sectional study, involving children aged 9–13 years and their parents, measuring dietary intake and predictors. For this analysis only, data from children aged 9–11 years were included to provide a sample comparable in age to participants in the RHS Project. Participants were recruited from grades five to seven of 27 primary schools in Adelaide. Participating schools distributed information to parents of students (n = 2575 children), with 1257 indicating consent (48.8% response rate), and 1201 students completing study measures. REACH was conducted over two phases: in phase one children completed a Child Nutrition Questionnaire (CNQ) about their dietary intake at school supported by fieldworkers, and in phase two parents completed a computer-assisted telephone interview (CATI). This analysis includes 347 participants aged 9–11 years with complete food intake (CNQ) and demographic (CATI) data.

### 2.2. RHS study methodology

The RHS School Gardening trial consisted of two parallel randomized controlled trials, to evaluate the effect of the school gardening program on children's fruit and vegetable intake [16]. Thirty-two schools from four London boroughs were recruited for trial two. For this analysis, baseline data from Trial two control schools which received no intervention were utilised. Diet was assessed using a modified version of the validated Child And Diet Evaluation Tool (CADET) food frequency questionnaire [28].

### 2.3. REACH study dietary assessment

The CNQ measuring children's dietary intake is a valid and reliable semi-quantitative food frequency questionnaire, completed in REACH as an online questionnaire [29]. For this analysis, data relating to children's breakfast, family meals and school-day food (recess (morning break time), lunch and after school not including dinner) were extracted. Children reported intake of commonly consumed foods and drinks at each time point (Table 1). Usual daily serves of fruit and vegetable intake were reported using a 5-point scale from “I don't eat fruit/vegetables” to “more than 5 serves per day”. For this analysis, the categories of “I don't eat fruit/vegetables” and “less than 1 serve per day” were combined. Frequency of breakfast intake was measured using one question; “*how often do you usually have something to eat for breakfast?*” on a 5-point Likert scale from never/rarely to everyday. Responses were dichotomised as “consumed breakfast every day” or “did not consume breakfast every day”. Family meal intake was measured using one question; “*how often do you eat dinner with most of the family?*” on a 5-point scale, which was categorised as “less than once a week”, “1–6 times per week” and “every day” for analysis.

### 2.4. RHS dietary assessment

The CADET diary comprised a list of 115 food and drink types within 15 categories. The CADET uses age and gender specific food portion sizes to calculate food and nutrient intake for children aged 3 to 11 years old [28],[30]. To complete the School and Home Food diaries, participants ticked each item consumed, under the appropriate meal time heading within the 24-h period. The School Food Diary was completed by a fieldworker at school for all school time meals, while the Home Food Diary was completed by parents at home. The Home Food Diary also included a question about family meal habits; “*on average, how many nights a week does your family eat at a table?*”. It was then categorized into tertiles; “less than once a week”, “1–6 times a week” and “every day” based on the distribution of the data and to categorise into broadly equal sized groups. Breakfast intake was dichotomised according to whether or not participants consumed items in the breakfast section. Consuming breakfast is linked to positive dietary behaviour, and cognitive function in children [31]. Children's fruit and vegetable consumption was measured in grams, however, for consistency between the two studies, fruit and vegetable serves were categorised into less than 1 serve per day, 1–2 serves per day, 3–5 serves per day or more than 5 serves per day. For this analysis a serve is equal to 80 grams of fruit or vegetables.

### 2.5. Data analysis

All analysis was conducted using STATA IC version12 [32]. The data were pooled then stratified by country for analysis. Differences in fruit and vegetable intake, school day food and drink intake between Australia and the UK were determined using chi-square analyses. Logistic regression was used to explore underlying differences between Australia and England for fruit, vegetables and at home meal behavior: breakfast consumption and evening meal consumption. The data were checked for possible clustering by school/class, using clustered multilevel regression models [30]. Clustering in schools was not evident so no adjustments were made. Demographic information was collected for child sex, child age, parent education, home postcode (as a proxy for neighbourhood disadvantage), and ethnicity, which were used as covariates in the analysis. These potential confounders were included in the models *a priori* based on a path analysis diagram created to explore factors which could assist or prevent the success of the intervention on the primary outcome [33]. These confounders have also been shown in the literature to be associated with children's dietary intake [34]. A *p*-value of less than 0.05 was taken to represent statistical significance for all analyses.

## 3. Results

Table 1 provides demographic characteristics of participants. Children's age and gender distributions were very similar in both groups. Problems with missing data were evident for parental education with the English sample having 54% data missing. Of those parents who reported their education levels, Australian children had a higher percentage of parents with low (Australian 28%, English 24%), mid (Australian 32%, English 22%), but not for high education (Australian 40%, English 53%). The English sample had more varied ethnicity, whereas more Australians predominantly identified as Caucasian. Breakfast intake was high in both groups (Australia 79%, English 93%). Due to the nature of food provision (hot meals provided in schools in England), there was a large variation in the percentages consuming packed lunch (Australia 90%, English 33%) and school meals(Australia 10%, English 67%). Family evening meal (eating a meal together at a table) consumption everyday was more frequent in Australia (68% compared to 34%), with a higher percentage of English children having a family evening meal less than once a week (30% compared to 9%).

**Table 1.** Demographics by Country.

	Australia (n = 347)	England (n = 425)
Child Characteristics		
Age (years; mean ± SD)	10.6 ± 0.5	9.7 ± 0.6
Boys [n (%)]	194 (56)	204 (48)
Ethnicity [n (%)]		
White	313 (90)	74 (17)
Mixed	2 (0.5)	47 (11)
Asian	26 (8)	42(10)
Black	6 (1.5)	85 (20)
Missing^#^	-	177 (42)
Parent Education [n (%)]		
High school or less	97 (28)	47 (24)
Trade or diploma	111 (32)	43 (22)
University degree or higher	139 (40)	104 (53)
Missing ˆ	-	231*
Meal type [n (%)]		
Packed lunch	305 (90)	135 (33)
School meal	34 (10)	273 (67)
Consumed breakfast	275 (79)	396 (93)
Family Meal [n (%)]		
Less than once a week	30 (9)	129 (30)
1–6 times a week	83 (24)	152 (36)
Every day	234 (68)	144 (34)
Fruit serves per day [n (%)]		
Less than 1 per day	42 (12)	105 (25)
1–2 serves per day	161 (46)	129 (30)
3–5 serves per day	102 (30)	126 (30)
More than 5 serves per day	42 (12)	65 (15)
Vegetable serves per day [n (%)]		
Less than 1 per day	8 (2)	72 (17)
1–2 serves per day	38 (11)	77 (18)
3–5 serves per day	158 (46)	161 (37)
More than 5 serves per day	143 (41)	115 (27)

^#^ Ethnicity data missing for 42 % of English participants (n = 177); percent distribution of ethnicity presented in table includes participants with missing data. Distribution of ethnicity without including missing data: White 30%, Mixed 19.0%, Asian 17%, Black 34%.

ˆ Education data missing for 54% English participants (n = 231); percent distribution of education presented in table only includes those who provided us with details.

- Means no missing data

There were considerable differences between foods and drinks consumed by Australian and English children during the school day (Table 2). The proportion of children from Australia consuming any food at recess was 77.8% (95% CI 73.4, 82.1) whereas for the English children it was less than half at only 45% (95% CI 40.8, 50.3). Australian children reported consuming significantly more water during recess and lunchtime, English children consumed more sweet drinks at lunch, all children consumed some food at lunchtime. Differences were observed in drinks consumed in the afterschool period, with more English children consuming water, milk and flavoured milk, whereas more Australian children consumed sweet drinks. The proportion of children who consumed drinks at either recess, lunch or after school was similar with Australian's consuming significantly more water (89.3% compared to 74.1%), milk/flavoured milk (23.0%. Compared to 15.5%) and fruit juice (38.6% compared to 29.2%), compared to English children, however there was no total difference in sweet drinks. Overall frequency of core foods consumed during the school day was similar, but the times at which foods were consumed differed between Australian and English children. Non-core foods, such as sweet biscuits and cakes, were consumed across all three meal events for Australian children; however, they were only consumed at lunchtime or after school among the English children. The proportion of Australian children who consumed these items at any point during the day was significantly more for potato crisps (52.2% compared to 24.7%), chocolate (27.4% compared to 2.4%), and lollies (18.2% compared to 16.5%), whereas English children consumed more biscuits and cakes (50.8% compared to 40%). Considerably more English children reported consuming vegetables at school than Australian children (recess/lunchtime Australian children 3.4%/6.1%; English children recess/lunchtime, 3.6/51.1%) and significantly more English children consumed vegetables in total for the school day (61.2% compared to 21.9%). For fruit, Australian children consumed more at recess (31.4% compared to 9.7%) whereas English children consumed more at lunchtime (Australian 17.6% compared to 27.2%). There was no significant difference in total number of children consuming fruit across the school day. The most commonly consumed fruit for both countries was an apple, and the most commonly consumed vegetables (not including potato) were carrots by Australian children, and peas and sweetcorn by English children (Table 3).

Additional analysis explored differences between Australian and English children for fruit and vegetable intake, family dinner and breakfast consumption (Table 4). Compared to English children there was a trend for Australian children to consume more vegetables per day in the unadjusted model. This relationship remained significant only for children who consumed more than five serves of vegetables per day after adjusting for possible confounders (OR = 4.1; 95% CI: 1.3, 12.5, *p* < 0.001). Whereas for fruit intake there were significant differences between countries for children who ate 1–2 (OR: 3.1; 95% CI: 2.0, 4.7, *p* < 0 .001) or 3–5 serves per day (OR: 2.0; 95% CI: 1.3, 3.1, *p* = 0.002). These differences did not remain significant once adjusted for possible confounders, however the overall trend analysis showed a significant difference. Compared to English children, Australian children were more likely to eat a family meal 1-6 times a week or every day in the unadjusted model (OR: 2.3; 95% CI: 1.4, 3.7, *p* < 0.001). The odds of having a family meal remains significant only for children who consumed it every day in the adjusted model (OR: 4.0; 95%CI: 1.8, 8.5, *p* < 0.001). However, for breakfast consumption, Australian children tended to be less likely to have breakfast than the English children. This was statistically significant in both unadjusted (OR: 0.2; 95% CI: 0.1, 0.5, *p* = 0.001) and adjusted models (OR: 0.2; 95% CI: 0.0, 0.7, *p* = 0.01).

**Table 2.** Consumption number (%) of foods and drinks during the school day at recess, lunch and afterschool (N = 772).

	Recess			Lunch			After school			Total intake (%)*		
	Aus	Eng	*p*	AUS	Eng	*p*	Aus	Eng	*p*	Aus	Eng	*p*
Drinks												
Water	226 (65.1)	32 (7.6)	<0.001	256 (73.8)	122 (28.7)	<0.001	197 (56.4)	256 (60.2)	0.331	310 (89.3)	315 (74.1)	<0.001
Milk/flavoured milk	9 (2.6)	0 (0.0)	<0.001	9 (2.6)	21 (4.9)	0.090	67 (11.3)	48 (19.3)	0.002	80 (23.0)	66 (15.5)	0.008
Fruit juice	31 (8.9)	11 (2.6)	<0.001	25 (7.2)	38 (8.9)	0.381	99 (28.5)	87 (20.5)	0.009	134 (38.6)	124 (29.2)	0.006
Sweet drinks^#^	13 (3.7)	1 (0.2)	<0.001	14 (4.0)	51 (12.0)	<0.001	102 (29.4)	90 (21.2)	0.009	111 (32.0)	132 (31.1)	0.770
Core foods												
Yoghurt	30 (8.7)	0 (0.0)	<0.001	9 (2.6)	118 (27.8)	<0.001	43 (12.4)	43 (10.1)	0.318	74 (21.3)	148 (34.8)	<0.001
Sandwich	10 (2.9)	0 (0.0)	<0.001	228 (65.7)	189 (44.5)	<0.001	42 (12.1)	99 (23.3)	<0.001	246 (70.8)	242 (56.9)	<0.001
Vegetables	13 (3.8)	0 (0.0)	<0.001	21 (6.1)	217 (51.1)	<0.001	56 (16.1)	68 (16.0)	0.958	76 (21.9)	260 (61.2)	<0.001
Fruit	109 (31.4)	41 (9.7)	<0.001	61 (17.6)	117 (27.5)	0.001	86 (24.8)	150 (35.3)	0.002	180 (51.9)	249 (58.6)	0.062
Dried fruit	9 (2.6)	3 (0.7)	0.035	2 (0.6)	0 (0.0)	0.117	11 (3.2)	4 (0.9)	0.026	15 (3.5)	15 (3.5)	0.780
Soup	2 (0.6)	0 (0.0)	-	5 (1.4)	0 (0.0)	-	18 (5.2)	0 (0.0)	-	21 (6.1)	0 (0.0)	-
Pasta/noodles	2 (0.6)	0 (0.0)	0.117	19 (5.5)	134 (31.5)	<0.001	40 (11.5)	45 (10.6)	0.678	60 (17.3)	164 (38.6)	<0.001
Non-core foods												
Hot chips/fries/wedges	3 (0.8)	0 (0.0)	0.055	6 (1.7)	68 (16.0)	<0.001	17 (4.9)	21 (5.1)	0.885	24 (6.9)	82 (20.1)	<0.001
Pizza	0 (0.0)	0 (0.0)	-	8 (2.3)	1 (0.2)	0.008	14 (4.0)	13 (3.1)	0.463	22 (6.3)	14 (4.0)	0.724
Pies/pasties/sausage roll/hot dog	2 (0.6)	0 (0.0)	0.117	12 (3.5)	28 (6.6)	0.051	22 (6.3)	16 (3.8)	<0.001	34 (9.8)	44 (10.4)	0.799
Potato crisps	151 (43.5)	0 (0.0)	<0.001	16 (3.4)	48 (11.1)	0.001	182 (52.5)	57 (16.4)	0.001	182 (52.4)	105 (24.7)	<0.001
Savoury biscuits/crackers	43 (12.4)	0 (0.0)	<0.001	19 (5.5)	52 (12.2)	0.001	43 (12.4)	72 (16.9)	0.077	87 (25.1)	113 (26.6)	0.632
Chocolates	61 (17.6)	0 (0.0)	<0.001	12 (3.5)	10 (2.4)	0.359	38 (11.0)	0 (0.0)	<0.001	95 (27.4)	10 (2.4)	<0.001
Lollies	25 (7.2)	0 (0.0)	<0.001	12 (3.5)	3 (0.7)	0.006	42 (12.1)	67 (15.8)	0.146	63 (18.2)	70 (16.5)	<0.001
Muesli bar	95 (27.4)	16 (3.8)	<0.001	25 (7.2)	21 (4.9)	0.186	28 (8.1)	18 (4.2)	0.025	131 (37.8)	52 (12.2)	0.537
Sweet biscuits/cakes/muffins	92 (26.5)	0 (0.0)	<0.001	14 (4.0)	113 (26.6)	<0.001	55 (15.9)	139 (32.7)	<0.001	136 (40.0)	216 (50.8)	0.001
Ice-cream	8 (2.3)	0 (0.0)	-	7 (2.0)	31 (7.3)	0.001	45 (13.0)	10 (2.4)	<0.001	55 (15.9)	41 (9.6)	0.549

^#^ Sweet drinks including soft drinks, cordial, energy drinks and soft drinks; *total intake of each item was only counted once per child. (*p* < 0.05) between Australian and English children.

**Table 3.** Types of fruits and vegetables consumed by children (N = 772)^a^.

	Aus	95% CI	Eng	95% CI	*p*
Fruit intake (%)					
Apple	70.3	55.7–84.2	31.7	15.7–44.2	<0.001
Banana	39.2	18.5–61.4	20.0	2.4–37.5	<0.001
Grapes	10.7	8.3–30.1	10.7	8.3–30.1	0.490
Kiwifruit	8.9	6.3–11.6	2.2	1.5–5.5	<0.001
Pear	17.3	6.2–40.2	7.4	1.8–22.8	<0.001
Pineapple	13.8	6.0–32.0	1.9	0.6–3.0	<0.001
Strawberries	30.0	11.6–48.3	6.6	4.0–7.9	<0.001
Citrus fruit (orange, mandarin)	24.9	3.6–36.3	1.2	0.1–30.4	<0.001
Melon (rock melon, watermelon)	40.1	15.5–61.4	19.4	2.4–37.5	<0.001
Vegetable intake (%)					
Broccoli, brussel sprouts and cabbage	38.6	20.6–55.3	7.3	2.1–16.1	<0.001
Capsicum	22.5	4.2–39.7	1.0	0.3–5.2	<0.001
Carrot	56.8	42.6–69.4	3.3	1.5–7.5	<0.001
Cauliflower	17.9	1.4–35.4	1.6	0.4–7.7	<0.001
Celery	12.1	1.4–38.0	6.4	1.3–25.0	0.004
Cucumber	31.4	13.8–48.1	2.2	0.3–7.9	<0.001
Legumes	13.8	7.8–33.8	14.8	7.5–35.5	0.696
Lettuce	38.6	20.0–55.9	10.6	2.6–46.8	<0.001
Peas and corn	43.8	22.7–63.2	19.3	2.9–35.0	<0.001
Potatoes	39.7	8.7–69.2	28.0	5.3–43.3	<0.001
Potatoes (fried)	25.9	3.0–46.9	10.1	0.5–25.1	<0.001
Spinach	7.8	1.3–27.4	0.7	0.0–7.3	<0.001
Tomato	32.3	12.9–51.0	7.3	3.4–17.4	<0.001
Other vegetables ˆ	47.3	29.1–64.8	17.2	3.5–30.4	<0.001

^a^ REACH study (AUS): Children reported whether or not they consumed fruit and vegetables on the day before data collection from a tick–list.

RHS study (England): The types of fruit and vegetables consumed were identified from the CADET diary.

**Table 4.** Likelihood for undertaking dietary behaviours in Australian compared to English children.

	Aus n	Eng n	OR	95% CI	*p*	*p* trend	OR	95% CI	*p*	*p* trend
Daily serves of vegetables						0.02				0.06
Less than 1 serving per day	8	72	REF				REF			
1–2 servings per day	38	77	4.4	1.9–10.1	<0.001		2.2	0.6–7.5	0.182	
3–5 servings a day	158	161	8.8	4.1–18.9	<0.001		2.7	0.9–8.1	0.063	
More than 5 servings per day	143	115	11.1	5.1–24.1	<0.001		4.1	1.3–12.5	0.011	
Daily serves of fruit						<0.001				<0.001
Less than 1 serving per day	42	105	REF				REF			
1–2 servings per day	161	129	3.1	2.0–4.7	<0.001		1.7	0.8–3.6	0.159	
3–5 servings a day	102	126	2.0	1.3–3.1	0.002		1.3	0.6–2.8	0.493	
More than 5 servings per day	42	65	1.6	0.9–2.7	0.075		0.6	0.2–1.6	0.416	
Frequency of family dinner						0.02				0.01
Less than once a week	30	129	REF				REF			
1–6 times per week	83	152	2.3	1.4–3.7	<0.001		0.9	0.4–2.1	0.949	
Every day	234	144	6.9	4.4–10.9	<0.001		4.0	1.8–8.5	0.001	
Frequency of breakfast intake						0.01				0.001
Did not consume	72	29	REF				REF			
Consumed breakfast	275	396	0.2	0.1–0.5	0.001		0.2	0.0–0.7	0.017	

Model 1–unadjusted model.

Model 2–model controlling for child sex, child age, ethnicity and parent education child sex, child age, ethnicity and parent education.

## 4. Discussion

This study investigated school day food consumption, breakfast intake and family meals in children from England and Australia, identifying potential country–specific targets for improving children's diets. Key findings highlighted differences in the types of foods consumed during the school day which may be related to the mode of school food provision (lunch box versus school meals). Compared to English children there was a trend for Australian children to consume more vegetables overall, despite consuming less vegetables at school. This is most likely to do with Australian children being more likely to have a family meal. Australian children were also more likely to consume discretionary snack foods during the school day. Conversely, English children were more likely to consume vegetables at school (51.1% versus 9.9%), provided in a hot school meal, such as curry or spaghetti. Due to food policies of many English schools where most children are not encouraged to have any food at recess, only 9.7% of English children consumed fruit at recess, compared with 32% of Australian children. The most commonly consumed fruits were apples, and bananas, two pieces of fruit that are easy to take to school and therefore an ideal snack.

These findings indicate that we have opportunities available to learn from existing policies and practices in each country in order to increase intake of fruit and vegetables and decrease intake of core foods within the school day as well as at home for children in Australia and England, but the targets for improvement differ for each country. In Australia where most children bring a packed lunch, improving the contents of packed lunches may improve vegetable intake at school, whereas in England where children consume more vegetables as part of a school lunch meal, improvements in vegetable provision in the home may be more beneficial for increasing vegetable intake.

Information from school based programmes provide an opportunity to share best practice between countries. School food provision and policies can play a fundamental role in influencing children's dietary intake and may provide a key leverage point for improving children's diets. [7],[19],[20], particularly for children of low socioeconomic status, where the home environment is less supportive of healthy eating. [35],[36]. Over recent years in England some primary schools have introduced salad bars, to help encourage children to consume vegetables, this works well for meals such as pizza, or jacket potatoes (two regular meals options in most primary schools) which contain few if any vegetables [37]. Freshly cut salad vegetables, could easily be sold in the Australia canteens, not just fruit.

The Crunch&Sip program [22] provided in some Australian primary schools, may provide an avenue to increase fruit intake in English schools. A simple sticker–based reward system run by some English schools encouraging children to eat seasonal fruits and vegetables, such as tropical fruits during summer, could improve children's school day intake of fruit and vegetables in both countries [38]. Despite higher intakes of fruit in Australia, this is dependent on socioeconomic status (SES), with low SES children consuming more discretionary foods and less fruit [27]. Selling pre–cut fruit and vegetables at school could be a sustainable method of increasing fruit and vegetable intake for primary school children in both countries. At lunchtime, a typical lunch meal in the UK is a hot meal, whereas a typical Australian school lunch is a sandwich provided from home. It is easier to include more variety and quantity of vegetables in a hot meal compared with a sandwich, where only 30 grams of cucumber or lettuces tends to be included according to CADET diaries [39]. However, introducing hot meals at lunchtime in Australia would involve a huge change in cultural traditions which may not be popular. Parents in Australia recognise the importance of providing a healthy lunchbox, but identify a number of barriers that prevent them from doing so, including convenience, child preferences, cost and food safety (lack of refrigeration) [40]. In the UK, Cooper et al. [41] conducted a study promoting healthy eating and meal ideas for school lunches, finding that packed lunches at follow–up included significantly fewer foods high in fat and sugar, fewer sweetened drinks, and more fruit and vegetables. The Smart Lunchbox randomised controlled trial conducted in the UK found moderate improvements in packed lunch quality, with children in the intervention group consuming on average more fruit and vegetables, dairy and starchy foods, and less savoury snack foods compared to the control group [42]. The effect of healthy school food programs may be diminished in the face of less supportive home food environments, which play an integral role in determining children's food intake. This is highlighted in studies showing that where free school meals are not provided, family–environment factors are more strongly related to children's fruit and vegetable intake [43]. Similarly, a recent systematic review found that school–based interventions which include a home–based element had the greatest effectiveness for obesity prevention [44].

Children's eating patterns are highly influenced by the types of food available to them at home, the types of food their parents consume, and rules around eating such as eating meals together as a family [35],[45]–[47]. A large European systematic review showed that breakfast intake may be protective against becoming overweight or obese [48]; Conversely, skipping breakfast has been associated with increased BMI in children and adolescents [48]–[50]. Dietary behaviour in the home is deeply rooted in cultural and social norms rather than shaped through policy and is likely therefore to be even harder to change than food during the school day. Eating a family meal together increases fruit and vegetable consumption [51] and may reduce risk of obesity [52]. Family meals are more common in Australia, but effective methods to increase family mealtimes in England remain elusive. Further qualitative research from both countries may provide valuable insights on this subject. As well as increasing the frequency of family meals, ways to improve the quality of family meals could also be implemented such as strategies to increase drinking water with meals rather than sugary drinks. One method could be to record what fruit and vegetables are consumed by family members and turn into a weekly competition [53]. Families should be provided with education around consuming fruit and vegetables in season to optimise budget and taste although in Australia it is likely that seasonal fruit is more varied due to differences in climate.

### 4.1. Strength and limitations

This novel comparison of children's dietary intake during the school day across two different school food provision systems, provides evidence for policy makers of how nutritious food intake for children may be supported during the school day. Whilst data utilised in this study is from 2010, more recent Australian and English 2012 national survey data indicate little improvement in children's diet quality, [54],[55] nevertheless the age of the data is a limitation [56]. It is worth nothing that an analysis of discretionary food intake in Australian children using data from the latest Australian National Nutrition survey showed that children's intake of discretionary foods intake has not changed from the last national survey in 2007, and continues to remain well above recommended intakes [56]. This study uses cross–sectional data and therefore causation cannot be determined. Further, the findings of the study cannot be generalised at the population level. All data were self–reported by the children at school with support by fieldworkers for the South–Australian study and at home by parents (afterschool, dinner and breakfast) for the England, London based study, therefore responses may be affected by misreporting or socially desirable response bias. The data collected during school hours in the England study was collected by trained field workers. In general, there are limitations in children's ability to accurately recall and report their dietary intake; however children of this age group are capable of self–reporting their dietary intake [57]. Both the CNQ used in the REACH study and the CADET diary used in the UK study have been shown to have good validity and reliability [29],[58]. The CADET study attempted to improve the quality of dietary data by providing parents and children with an instruction DVD explaining how to complete the food diary. It can also explore dietary intake of fruit and vegetables from meals combined together, such as intake from curries, pies and other foods their intake is included in the total intake. The diary has also been validated in a South–Asian population, to make it accessible for the different ethnic minority groups that represent the English population [59]. The REACH study child questionnaires were administered online allowing for in–built measures such as forced question responses, which reduced missing data and errors associated with data entry.

## 5. Conclusion

By comparing children's school day food intake across two countries with different school food provision practices, this study provides evidence of how modes of school food provision may influence children's food intake. Findings indicate that provision of a school lunch meal, compared to a packed lunch from home, may be more supportive of children's vegetable intake for that meal. However, without a supportive home environment that encourages vegetable intake, children will not be able to consume sufficient amounts of vegetables. This comparison of data from England and Australia shows that although overall the food intake of children in both countries needs to be improved, the targets for improvement differ in each country. In Australia, efforts are needed to support parents to improve the nutritional quality of lunchboxes. Conversely, in England school meals appear to support healthy food intake during the school day, however children had poorer dietary behaviors at home, suggesting the dietary intervention in England should be more focused on improving vegetable provision, breakfast intake and family meals in the home.
