# Correction: Comparison of school day eating behaviours of 8–11 year old children from Adelaide, South Australia, and London, England: Child eating behaviours in South Australia and England

**DOI:** 10.3934/publichealth.2018.4.477

**Published:** 2019-01-03

**Authors:** Dorota Zarnowiecki, Meaghan S Christian, James Dollman, Natalie Parletta, Charlotte E.L Evans, Janet E Cade

**Affiliations:** 1 School of Pharmacy and Medical Sciences, University of South Australia; City East Campus, North Terrace, Adelaide, SA 5000; 2 School of Clinical and Applied Sciences, Faculty of Health and Social Sciences, Leeds Beckett University; City Campus, Calverley Street, Leeds, LS1 3HE, UK; 3 Nutritional Epidemiology Group, School of Food Science and Nutrition, University of Leeds; Woodhouse Lane, Leeds, LS2 9JT, UK; 4 Alliance for Research in Exercise, Nutrition and Activity, School of Health Sciences, University of South Australia; City East Campus, North Terrace, Adelaide, SA 5000; 5 Centre for Population Health, School of Health Sciences, University of South Australia; South Australian Health and Medical Research Institute (SAHMRI), North Terrace, Adelaide, SA 5000

## A correction on

Comparison of school day eating behaviours of 8–11 year old children from Adelaide, South Australia, and London, England

Running title: Child eating behaviours in South Australia and England

by Dorota Zarnowiecki, Meaghan S Christian, James Dollman, Natalie Parletta, Charlotte E.L Evans, Janet E Cade. AIMS Public Health , 2018, 5(4): 394-410. doi: 10.3934/publichealth.2018.4.394

We would like to submit the following corrections to our recently published paper [1] due to the wrong version of the manuscript. The details are the following.

1. The first paragraph in section 1 has been updated.

“The impact of poor nutrition in children is causing public health concerns around the world, and contributing to rising childhood obesity [1,2]. Diet plays a fundamental role in weight management, and it is vital to foster healthy dietary behaviours from a young age to establish long term healthy habits [1,3,4]. Recently Evans et al have explored how the policies around health promotion and provision of food during the school day are having a considerable impact on overall diet intake in children [5]. Two countries with considerably different school food provision practices are Australia and England. England school food provision is centred on the school providing a school meal, whereas in Australia, most children bring in their own meal prepared by their parents, a “packed lunch” [6]. Only one other study has considered how differing food provision practices affect children's overall nutritional intake in England and Australia [7]. This review highlighted considerable differences in school nutrition policies between Australian and England, noting potential nutritional advantages of school meal provision for improving diet quality in children. However, understanding of the potential impact of school nutrition policies for improving nutritional intake is limited by a lack of monitoring and evaluation of policy compliance and dietary intake. Another important finding was whilst during school hours children's food consumption is restricted to meet nutrition standards it is the home food environment that can have a lasting impact on overall diet quality [7].”

2. The table 1 has been updated.

**Table 1.** Demographics by Country.

	Australia (n = 347)	England (n = 425)
Child Characteristics		
Age (years; mean ± SD)	10.6 ± 0.5	9.7 ± 0.6
Boys [n (%)]	194 (56)	204 (48)
Ethnicity [n (%)]		
White	313 (90)	74 (17)
Mixed	2 (0.5)	47 (11)
Asian	26 (8)	42(10)
Black	6 (1.5)	85 (20)
Missing^#^	-	177 (42)
Parent Education [n (%)]		
High school or less	97 (28)	47 (24)
Trade or diploma	111 (32)	43 (22)
University degree or higher	139 (40)	104 (53)
Missing ˆ	-	231*
Meal type [n (%)]		
Packed lunch	305 (90)	135 (33)
School meal	34 (10)	273 (67)
Consumed breakfast	275 (79)	396 (93)
Family Meal [n (%)]		
Less than once a week	30 (9)	129 (30)
1–6 times a week	83 (24)	152 (36)
Every day	234 (68)	144 (34)
Fruit serves per day [n (%)]		
Less than 1 per day	42 (12)	105 (25)
1–2 serves per day	161 (46)	129 (30)
3–5 serves per day	102 (30)	126 (30)
More than 5 serves per day	42 (12)	65 (15)
Vegetable serves per day [n (%)]		
Less than 1 per day	8 (2)	72 (17)
1–2 serves per day	38 (11)	77 (18)
3–5 serves per day	158 (46)	161 (37)
More than 5 serves per day	143 (41)	115 (27)

^#^ Ethnicity data missing for 42 % of English participants (n = 177); percent distribution of ethnicity presented in table includes participants with missing data. Distribution of ethnicity without including missing data: White 30%, Mixed 19.0%, Asian 17%, Black 34%.

ˆ Education data missing for 54% English participants (n = 231); percent distribution of education presented in table only includes those who provided us with details.

- Means no missing data

3. The second paragraph in section 3 has been updated.

“There were considerable differences between foods and drinks consumed by Australian and English children during the school day (Table 2). The proportion of children from Australia consuming any food at recess was 77.8% (95% CI 73.4, 82.1) whereas for the English children it was less than half at only 45% (95%CI 40.8, 50.3). Australian children reported consuming significantly more water during recess and lunchtime, English children consumed more sweet drinks at lunch, all children consumed some food at lunchtime. Differences were observed in drinks consumed in the afterschool period, with more English children consuming water, milk and flavoured milk, whereas more Australian children consumed sweet drinks. The proportion of children who consumed drinks at either recess, lunch or after school was similar with Australian's consuming significantly more water (89.3% compared to 74.1%), milk/flavoured milk (23.0%. Compared to 15.5%) and fruit juice (38.6% compared to 29.2%), compared to English children, however there was no total difference in sweet drinks. Overall frequency of core foods consumed during the school day was similar, but the times at which foods were consumed differed between Australian and English children. Non-core foods, such as sweet biscuits and cakes, were consumed across all three meal events for Australian children; however, they were only consumed at lunchtime or after school among the English children. The proportion of Australian children who consumed these items at any point during the day was significantly more for potato crisps (52.2% compared to 24.7%), chocolate (27.4% compared to 2.4%), and lollies (18.2% compared to 16.5%), whereas English children consumed more biscuits and cakes (50.8% compared to 40%). Considerably more English children reported consuming vegetables at school than Australian children (recess/lunchtime Australian children 3.4%/6.1%; English children recess/lunchtime, 3.6/51.1%) and significantly more English children consumed vegetables in total for the school day (61.2% compared to 21.9%). For fruit, Australian children consumed more at recess (31.4% compared to 9.7%) whereas English children consumed more at lunchtime (Australian 17.6% compared to 27.2%). There was no significant difference in total number of children consuming fruit across the school day. The most commonly consumed fruit for both countries was an apple, and the most commonly consumed vegetables (not including potato) were carrots by Australian children, and peas and sweetcorn by English children (Table 3).”

4. The table 2 has been updated.

5. One reference has been added.

Rampersaud GC (2009) Benefits of breakfast for children and adolescents: Update and recommendations for practitioners. *Am J Lifestyle Medicine* 3: 86–103.

**Table 2.** Consumption number (%) of foods and drinks during the school day at recess, lunch and afterschool (N = 772).

	Recess			Lunch			After school			Total intake (%)*		
	Aus	Eng	*p*	AUS	Eng	*p*	Aus	Eng	*p*	Aus	Eng	*p*
Drinks												
Water	226 (65.1)	32 (7.6)	<0.001	256 (73.8)	122 (28.7)	<0.001	197 (56.4)	256 (60.2)	0.331	310 (89.3)	315 (74.1)	<0.001
Milk/flavoured milk	9 (2.6)	0 (0.0)	<0.001	9 (2.6)	21 (4.9)	0.090	67 (11.3)	48 (19.3)	0.002	80 (23.0)	66 (15.5)	0.008
Fruit juice	31 (8.9)	11 (2.6)	<0.001	25 (7.2)	38 (8.9)	0.381	99 (28.5)	87 (20.5)	0.009	134 (38.6)	124 (29.2)	0.006
Sweet drinks^#^	13 (3.7)	1 (0.2)	<0.001	14 (4.0)	51 (12.0)	<0.001	102 (29.4)	90 (21.2)	0.009	111 (32.0)	132 (31.1)	0.770
Core foods												
Yoghurt	30 (8.7)	0 (0.0)	<0.001	9 (2.6)	118 (27.8)	<0.001	43 (12.4)	43 (10.1)	0.318	74 (21.3)	148 (34.8)	<0.001
Sandwich	10 (2.9)	0 (0.0)	<0.001	228 (65.7)	189 (44.5)	<0.001	42 (12.1)	99 (23.3)	<0.001	246 (70.8)	242 (56.9)	<0.001
Vegetables	13 (3.8)	0 (0.0)	<0.001	21 (6.1)	217 (51.1)	<0.001	56 (16.1)	68 (16.0)	0.958	76 (21.9)	260 (61.2)	<0.001
Fruit	109 (31.4)	41 (9.7)	<0.001	61 (17.6)	117 (27.5)	0.001	86 (24.8)	150 (35.3)	0.002	180 (51.9)	249 (58.6)	0.062
Dried fruit	9 (2.6)	3 (0.7)	0.035	2 (0.6)	0 (0.0)	0.117	11 (3.2)	4 (0.9)	0.026	15 (3.5)	15 (3.5)	0.780
Soup	2 (0.6)	0 (0.0)	-	5 (1.4)	0 (0.0)	-	18 (5.2)	0 (0.0)	-	21 (6.1)	0 (0.0)	-
Pasta/noodles	2 (0.6)	0 (0.0)	0.117	19 (5.5)	134 (31.5)	<0.001	40 (11.5)	45 (10.6)	0.678	60 (17.3)	164 (38.6)	<0.001
Non-core foods												
Hot chips/fries/wedges	3 (0.8)	0 (0.0)	0.055	6 (1.7)	68 (16.0)	<0.001	17 (4.9)	21 (5.1)	0.885	24 (6.9)	82 (20.1)	<0.001
Pizza	0 (0.0)	0 (0.0)	-	8 (2.3)	1 (0.2)	0.008	14 (4.0)	13 (3.1)	0.463	22 (6.3)	14 (4.0)	0.724
Pies/pasties/sausage roll/hot dog	2 (0.6)	0 (0.0)	0.117	12 (3.5)	28 (6.6)	0.051	22 (6.3)	16 (3.8)	<0.001	34 (9.8)	44 (10.4)	0.799
Potato crisps	151 (43.5)	0 (0.0)	<0.001	16 (3.4)	48 (11.1)	0.001	182 (52.5)	57 (16.4)	0.001	182 (52.4)	105 (24.7)	<0.001
Savoury biscuits/crackers	43 (12.4)	0 (0.0)	<0.001	19 (5.5)	52 (12.2)	0.001	43 (12.4)	72 (16.9)	0.077	87 (25.1)	113 (26.6)	0.632
Chocolates	61 (17.6)	0 (0.0)	<0.001	12 (3.5)	10 (2.4)	0.359	38 (11.0)	0 (0.0)	<0.001	95 (27.4)	10 (2.4)	<0.001
Lollies	25 (7.2)	0 (0.0)	<0.001	12 (3.5)	3 (0.7)	0.006	42 (12.1)	67 (15.8)	0.146	63 (18.2)	70 (16.5)	<0.001
Muesli bar	95 (27.4)	16 (3.8)	<0.001	25 (7.2)	21 (4.9)	0.186	28 (8.1)	18 (4.2)	0.025	131 (37.8)	52 (12.2)	0.537
Sweet biscuits/cakes/muffins	92 (26.5)	0 (0.0)	<0.001	14 (4.0)	113 (26.6)	<0.001	55 (15.9)	139 (32.7)	<0.001	136 (40.0)	216 (50.8)	0.001
Ice-cream	8 (2.3)	0 (0.0)	-	7 (2.0)	31 (7.3)	0.001	45 (13.0)	10 (2.4)	<0.001	55 (15.9)	41 (9.6)	0.549

^#^ Sweet drinks including soft drinks, cordial, energy drinks and soft drinks; *total intake of each item was only counted once per child. (*p* < 0.05) between Australian and English children.
